# Three-Dimensional Motion Perception: Comparing Speed and Speed Change Discrimination for Looming Stimuli

**DOI:** 10.3390/vision4030033

**Published:** 2020-07-06

**Authors:** Abigail R. I. Lee, Justin M. Ales, Julie M. Harris

**Affiliations:** 1 Centre for Vision Research, York University, Toronto, ON M3J 1P3, Canada; 2 School of Psychology and Neuroscience, University of St Andrews, St Andrews KY16 9JP, UK; jma23@st-andrews.ac.uk (J.M.A.); jh81@st-andrews.ac.uk (J.M.H.)

**Keywords:** looming, motion in depth, speed discrimination, speed change discrimination

## Abstract

Judging the speed of objects moving in three dimensions is important in our everyday lives because we interact with objects in a three-dimensional world. However, speed perception has been seldom studied for motion in depth, particularly when using monocular cues such as looming. Here, we compared speed discrimination, and speed change discrimination, for looming stimuli, in order to better understand what visual information is used for these tasks. For the speed discrimination task, we manipulated the distance and duration information available, in order to investigate if participants were specifically using speed information. For speed change discrimination, total distance and duration were held constant; hence, they could not be used to successfully perform that task. For the speed change discrimination task, our data were consistent with observers not responding specifically to speed changes within an interval. Instead, they may have used alternative, arguably less optimal, strategies to complete the task. Evidence suggested that participants used a variety of cues to complete the speed discrimination task, not always solely relying on speed. Further, our data suggested that participants may have switched between cues on a trial to trial basis. We conclude that speed changes in looming stimuli were not used in a speed change discrimination task, and that naïve participants may not always exclusively use speed for speed discrimination.

## 1. Introduction

Perceiving the speed of objects moving towards us in the world is important in our daily lives, for example when safely crossing a road. Of particular importance is the ability to judge the speed, and speed changes, of objects approaching in three dimensions. There are both monocular and binocular sources of visual information we can use to judge these movements. Speed discrimination using binocular cues to motion in depth has been well studied [1,2,3,4,5,6,7]. Perhaps more overlooked recently is the contribution of monocular cues to motion in depth, such as looming. Looming is usually defined as the change in retinal size that occurs when an object moves towards or away from an observer (e.g., [8]). This is the definition we use here. The first evidence for the existence of mechanisms specifically sensitive to such change in size, which could be used for the perception of motion-in-depth, came from motion adaptation studies demonstrating that adaptation to size-change was separable to that for lateral motion [9,10].

In this study, we investigated speed and speed change discrimination for looming stimuli, and explored the strategies that naïve participants may be using for speed discrimination. We define looming as the expansion of the image of an object on the retina as it approaches, while the object in the world remains a constant size. When an object approaches or moves away from an observer at a constant speed in the world, the image of that object on the retina accelerates or decelerates, respectively, as well as growing or shrinking in size. The closer the object gets to the eye, the greater the acceleration. Therefore, to emulate real-world motion, our looming stimuli moved at a constant world speed, which resulted in an accelerating retinal speed (see [11]).

Looming is thought to play a role in judging the speed of objects moving towards us. Speed discrimination thresholds for looming stimuli can be as low as 5% [8], similar to those for 2D motion [12,13,14,15,16,17,18]. By comparison, speed discrimination thresholds when using binocular cues to motion in depth are often much higher than that reported for looming and for 2D motion stimuli, indicating poorer performance [1,2,5,7]. The improved thresholds for looming cues over binocular cues suggests that looming is a critical cue for making judgements about the 3D motion of objects.

However, speed discrimination tasks can be problematic to interpret. In traditional speed discrimination designs, where participants view two stimulus speeds in either one or two temporal intervals and indicate which of the two is faster, it is impossible to be certain that participants judge speed, rather than distance or duration. Typically, if distance is kept constant, a participant could use speed or duration to make judgements. Conversely, if duration is held constant, participants can use speed or distance to make their judgements (for a review, see [19]). To avoid this problem, speed change discrimination tasks have been developed [16,20]. In these tasks, observers are asked to identify which of two temporal intervals contains a speed change, allowing total duration and distance to be held constant. In the standard interval, a stimulus travels at a constant speed. In the test interval, the stimulus first travels slower and then an equal amount faster than the standard interval speed. Thus, the mean speed, and therefore the duration and distance, are kept constant whilst speed is varied. There is evidence from studies that explore 2D motion, and several types of 3D motion, that speed change discrimination is much more difficult than speed discrimination (e.g., [11,16,20]).

No study has previously explored whether speed change discrimination for the monocular 3D motion cue of looming is more difficult than speed discrimination, and if so, why this may be. However, it is possible that speed discrimination may be an easier task because participants are able to use additional distance or duration cues to give their responses. We therefore had two aims:

(1) To determine if speed change discrimination for looming is a more difficult task than speed discrimination.
(2) To determine if participants use distance or duration information, rather than speed information, when it is available in speed discrimination tasks.

To address these aims, we used three experimental conditions, one in which we measured speed change discrimination, and two where we measured speed discrimination, but for stimuli that could either have duration cues, or distance cues, in addition to speed. For these conditions, we describe looming in terms of the distance and speed travelled in depth in the real world.

## 2. Methods

### 2.1. Participants

Participants were required to have normal or corrected-to-normal vision and a stereoacuity of at least 120 arcseconds, as measured by the TNO test (16th edition). For the speed change discrimination task, we recruited 15 participants. Two participants had a stereoacuity of over 120 arcseconds, 1 participant was unable to do the task, and 1 participant stopped attending the testing sessions, leaving 11 participants who completed the experiment (8 female, 3 male, aged between 18 and 34). For the speed discrimination tasks, we recruited 9 new naive participants, as the participants from the speed change discrimination task could not be recalled in line with our ethical approval requirements. One participant from this new group had a stereoacuity of over 120 arcseconds, whilst another participant did not pass the training, leaving 7 participants who completed the experiment (5 female, 2 male, aged between 18 and 28). Participants gave informed consent before beginning the experiment and all procedures were approved by the University of St Andrews University Teaching and Research Ethics Committee (UTREC; Approval code: PS11904). All experiments adhered to the tenets of the Declaration of Helsinki.

### 2.2. Materials

Stimuli were presented on an Iiyama MM904UTA Vision Master Pro 455 cathode ray tube screen with a refresh rate of 85 Hz and a resolution of 1280 × 1024 using a MacPro. A Cambridge Research Systems ColorCal MK II colorimeter was used to calibrate screen luminance, and an accurate pixel per centimetre conversion was obtained by measuring lines on the screen by hand. The screen was viewed through a 4-mirror stereoscope (because the set-up was also used for concurrent binocular vision experiments). However, here, the right and left eyes’ views were always identical (zero binocular disparity). Including the distances between the stereoscope mirrors, participants viewed the screen from a distance of 97 cm. All data, stimulus presentation code, and analysis code is available online at https://osf.io/xvs5n/.

### 2.3. Experiment Design

#### 2.3.1. Main Experiment Stimulus Design

**Figure 1:**
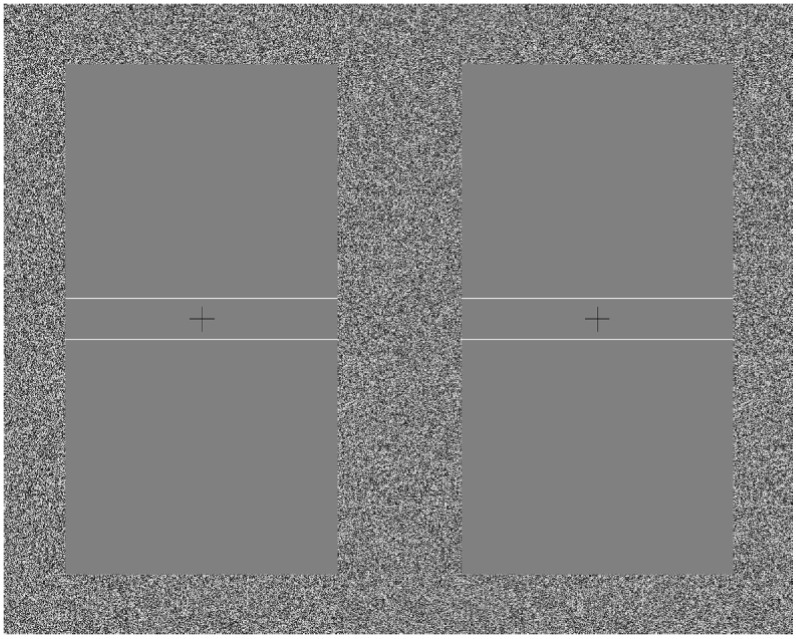
The looming stimulus used for all experimental conditions: a pair of horizontal white lines, moving on a grey background. The bottom lines moved downwards and the top lines moved upwards to simulate looming motion towards the observer. The left and right halves of the screen were delivered to each eye separately via a 4-mirror stereoscope to deliver a fused percept, but there was no binocular disparity displayed in the stimulus.

All stimuli were created using MATLAB R2014b (The MathWorks Inc., Natick, MA, USA) and Psychtoolbox 3 [21,22,23]. Stimuli were presented on a grey background with a luminance of 29.9 cd/m^2^. The stimulus used in the main experimental conditions consisted of a pair of white horizontal lines that were 6.96 degrees long and had a luminance of 59.9 cd/m^2^ (see Figure 1). The horizontal lines in the stimulus expanded away from one another in opposite directions (up/down on screen) to deliver the looming cue. Sekuler [8] suggests that looming is encoded by the pooling of unidirectional motion signals. Each of the two horizontal lines in the stimulus can be considered an independent set of pooled unidirectional motion signals. The two lines have opposing motion directions, generating a looming signal. Horizontal lines were used as they create no horizontal disparity, so that there would be no conflict between the looming cues and the lack of changing binocular disparity. The pair of lines simulated a very wide object approaching the observer (whose left and right edges were outside the field of view). Each line remained a constant 0.95 arcmin wide on the screen. The separation between the lines varied over time to simulate motion towards the observer, but at the plane of fixation (the screen distance of 97 cm), the separation between the two lines was 2 cm. The starting separation of the two lines was therefore 2 cm for the speed discrimination conditions where line movement began at the plane of fixation, and 1.66 cm for the speed change discrimination condition where line movement began from 20 cm behind the plane of fixation.

**Figure 2:**
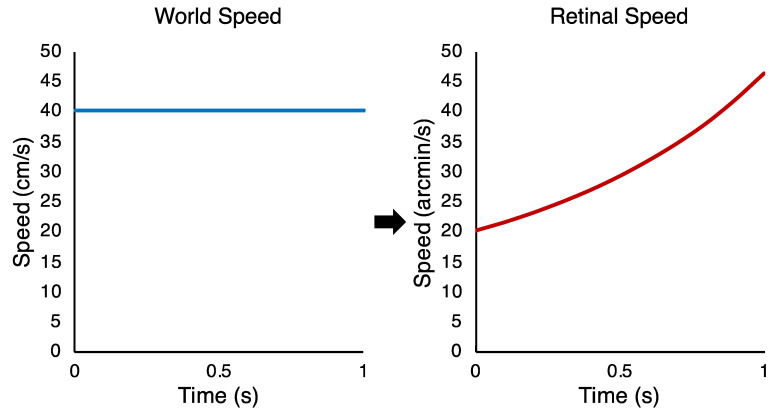
Speed–time diagrams of how an object moving towards an observer at 40 cm/s in the world, from a starting distance of 117 cm from the eye (**left**), translates into an accelerating retinal speed for the expansion of the object’s retinal image (**right**).

To emulate real-world motion, the two lines that comprised the stimulus for the main experimental conditions travelled at a constant world speed in depth. This translates into an accelerating speed on the retina (see Figure 2). This occurs because the retinal position of the stimulus is inversely proportional to its instantaneous distance from the observer. As the retinal speed is the derivative of retinal position, this means the retinal speed is not constant for a constant world speed (for further detail, see [11]). Note that when we refer to distances and speeds of motion in the text below, we refer to motions in the world. In this study, all of those world motions were along the depth dimension.

Stimuli were presented with a central black fixation cross with a luminance of 0.09 cd/m^2^. This was 37.9 long by 37.9 arcmin wide. To indicate when a response was required, a black 56.9 by 56.9 arcmin box of the same luminance appeared around the fixation cross. The fixation cross and box had line widths of 0.95 arcmin. Throughout each trial, an aperture frame of approximately uniformly distributed luminance noise, with individual pixels randomly assigned grey levels, was displayed around all stimuli. The aperture frame was 1.58 degrees wide, and had a minimum luminance of 0.09 cd/m^2^ and a maximum luminance of 59.9 cd/m^2^. The half-screen visible through the stereoscope used was 10.1 degrees wide and 16.2 degrees tall. Within the aperture frame, a rectangle 6.96 degrees wide and 13.0 degrees tall was used for stimulus presentation.

#### 2.3.2. Main Experiment Conditions

The stimulus used contained constant world speed (accelerating retinal speed) for three main experimental conditions:

(I) *Speed change discrimination* containing no useful distance or duration information. The task was to judge which interval contained a speed change.
(II) *Duration (speed discrimination*) containing duration and speed information. The task was to discriminate which interval contained faster motion. Either speed or duration information could be used to complete the task.
(III) *Distance (speed discrimination)* containing distance and speed information. The task was to again discriminate which interval contained faster motion. Either speed or distance information could be used to complete the task.

We define the looming in these conditions in terms of the speed and distance travelled in depth in the real world, rather than as a size change on the screen.

#### 2.3.3. Training Stimuli

Two further stimuli were used only for training purposes before the main experiment began: Training I: a square drifting grating with a spatial frequency of 1 cycle per degree that moved from left to right at a constant retinal speed. The grating was 4 degrees tall and 4 degrees wide. Training II: a pair of white vertical lines with a luminance of 59.9 cd/m^2^ that moved from left to right at a constant retinal speed. These lines were each 13.0 degrees tall and 0.95 arcmin wide.

### 2.4. Procedure

#### 2.4.1. Speed Change Discrimination

For the *Speed change* discrimination condition, participants completed a 2-interval forced choice task, with a 7-level method of constant stimuli design. One interval contained an instantaneous speed change from a slower to a faster speed, the other contained motion at a constant speed. Participants were asked to identify the interval that contained the speed change. Each interval began with the stimulus appearing, rendered 20 cm behind the plane of fixation (117 cm from the observer), and remaining stationary for 250 ms, before moving for 1 s. If the interval contained a change in speed, it occurred after 500 ms of motion. There was a gap of 1 s between the two intervals. Before the first interval, participants heard one beep; before the second, they heard two beeps. A run consisted of 210 trials divided into 3 blocks, each with 10 trials per level, giving 70 trials per block. These blocks were presented in a random order amongst those for a different study, with other speed change blocks containing either binocular or binocular and looming cues to motion in depth.

A standard speed of 40 cm/s towards the observer was used, which translated to a speed range of 20.1–46.3 arcmin/s on the retina over the full interval. In each successive level of the condition, the speed before the speed change decreased by 5 cm/s, and the speed after the change increased by 5 cm/s in the world. This meant that the test speeds were (speed before change–speed after change): 40–40, 35–45, 30–50, 25–55, 20–60, 15–65, and 10–70 cm/s. In the maximum speed change level, from 10 to 70 cm/s, this translates into a range of retinal speeds between 5.02 and 5.48 arcmin/s for the period before the instantaneous speed change, and a range of retinal speeds between 38.4 and 80.9 arcmin/s after the change.

All participants were required to complete three training blocks prior to the main experiment, which used the same task, but different stimuli. The Training I stimulus, a drifting grating, was used first in a speed change discrimination task like that used in the main experiment, and participants received audio feedback on their responses. The second block was identical, except participants no longer received feedback. The third block used the Training II stimulus (two vertical lines) in a speed change discrimination task with no feedback. Stimuli in these 3 training blocks all used constant retinal speed and contained the same 2 speed change levels. Participants compared a standard stimulus moving at 283.4 arcmin/s to a step speed change from 212.6 to 354.1 arcmin/s, or from 70.9 to 495.3 arcmin/s. The purpose of the training blocks was to introduce participants to the task by first using a commonly-used motion stimulus undergoing lateral motion (Training I), then a stimulus more similar to that used in the main experiment (Training II).

#### 2.4.2. Speed Discrimination with Duration or Distance

For the speed discrimination conditions containing either duration or distance cues (*Duration* and *Distance*), participants viewed two temporal intervals that both contained stimuli travelling at a constant world speed. The task was to pick the interval in which the stimulus moved faster. Participants were told to judge the speed, but were not given any instructions about use of distance or duration information. For each condition, two consistent cues were available (either speed and duration, or speed and distance). Our aim was to test whether performance was better for one condition or the other and compare these speed discrimination conditions to the *Speed change* condition (where only speed could be used). In the *Duration* and *Distance* conditions, catch trials were also included (see Section 2.4.3). A 7-level method of constant stimuli design was used. Each interval began with the stimulus appearing at the plane of fixation (97 cm from the observer) and remaining stationary for 0.250 s. The *Distance (speed discrimination)* condition, where duration was fixed but distance information was available, contained 0.506 s of motion in both the standard and test intervals. Each stimulus speed presented had a single stimulus distance associated with it, and the stimulus distance was not jittered. The distances presented ranged between 20 cm in depth in the standard speed level and 35 cm in depth in the maximum speed level and 42.8 cm in the catch interval. The *Duration (speed discrimination)* condition, where distance was fixed but duration information was available, consisted of a test interval containing a range of durations to keep the distance travelled constant at 28.7 cm towards the observer. Here, each stimulus speed presented had a single stimulus duration associated with it, and the stimulus duration was not jittered. The durations ranged between 0.412 s of motion for the maximum speed level, and 0.717 s of motion for the minimum speed level. The standard interval in the *Duration* condition was presented for 0.717 s. There was a gap of 1 s between intervals, and before the first interval, participants heard one beep; before the second, they heard two beeps. The duration of the catch interval was always 1.070 s, and the standard interval duration was the same as for the main experimental trials in that block (0.717 s for *Duration* and 0.506 s for *Distance*). The *Duration* and *Distance* conditions each consisted of 210 main experiment trials, which were randomly interleaved with the catch trials presented in 3 blocks with 10 trials per level, to provide participants with breaks. Blocks contained either only *Duration* or only *Distance* trials, but blocks of each condition were presented in a random order.

In both speed discrimination conditions, the standard stimulus had a speed of 40 cm/s in the world. Successive test levels increased in speed by 5 cm/s, to a maximum speed of 70 cm/s. This meant that test levels had speeds of 40 cm/s, 45 cm/s, 50 cm/s, 55 cm/s, 60 cm/s, 65 cm/s, and 70 cm/s. We chose these speeds so that they would be matched to the second speeds used in the test intervals of the *Speed change* discrimination condition (see above). In the *Distance* condition stimuli, these translated into retinal speeds of 29.2–46.3 arcmin/s in the standard level, and 51.2–125.0 arcmin/s in the maximum speed level. In the *Duration* condition stimuli, these world speeds translated into retinal speeds of 29.2–58.8 arcmin/s in the standard level, and 51.2–103.7 arcmin/s in the maximum speed level. In the catch trials, the speed of stimuli in the catch and standard intervals was 40 cm/s in the world, which translates differently into accelerating retinal speeds depending on the duration of the interval. In the *Duration* condition, the standard interval stimulus speeds ranged between 29.2 and 58.8 arcmin/s, and the catch interval speeds ranged between 29.2 and 93.4 arcmin/s. In the *Distance* condition, the stimulus speeds in the standard interval ranged between 29.2 and 46.6 arcmin/s, while the catch interval speeds ranged between 29.2 and 93.4 arcmin/s.

Prior to the experiment start, participants completed two speed discrimination training blocks, one *Duration* and one *Distance*, using the horizontal line stimulus used in the main experiment. Participants received audio feedback on their responses, and two levels of speed difference were used. The standard stimulus moved towards the observer at 40 cm/s, whilst the test stimulus had a speed of 50 cm/s or 70 cm/s in the world.

#### 2.4.3. Determining which Cue is Used for Speed Discrimination with Catch Trials

For each of the two speed discrimination conditions (*Distance* and *Duration*), we included a total of 30 “catch trials” that were designed to reveal what cues participants were using to perform the task. These trials contained standard and catch *intervals*. The catch intervals contained the same speed as the standard interval but had increased distance and duration (something travelling for longer at the same speed travels further). The duration of the catch interval was always 1.070 s, and the standard interval duration was the same as for the main experimental trials in that block (0.717 s for *Duration* and 0.506 for *Distance*). This difference results in catch intervals that should be easily discriminable if the participants were using distance or duration cues.

We coded a participant’s response as “correct” if they chose the catch interval and “incorrect” if they chose the standard. Because the catch trials contained the same speed in both intervals, if participants used speed only, we would expect performance to be at 50%. However, the catch intervals contained increased distance travelled and duration compared to the standard. Increased distance travelled might be associated with an object appearing to travel faster. If so, participants should choose the catch interval more often. An increased duration might be associated with an object appearing to travel slower. If using duration, participants would choose the standard interval more often. No matter what rule the participant used, if either distance or duration was used in addition to, or instead of speed to perform the task, we would expect performance to be different from 50% for these catch trials.

We made a simple assumption—That participants would use only one cue (duration, distance, or speed), and they would attempt to use the same cue across all trials. Here, we had a null hypothesis that if people used solely speed information, they would be picking the catch trial 50% of the time, because the catch and the standard intervals contain the same speed. We used the binomial test to determine if we could reject the null hypothesis. To do this, we used the binomial distribution to find which values were outside of the 95% confidence interval for the null hypothesis. Values outside of the confidence intervals indicated that we should reject the null hypothesis, and suggested that participants were using a cue other than speed to complete the task. For our 30 catch trials per condition, the 95% binomial proportion confidence interval for 50% performance was between 30% and 70%. Thus, if people picked the catch 21 or more times (70% of occasions or more), we rejected the null hypothesis and inferred that participants were not solely using speed. If the catch interval was picked 9 or less times (30% of occasions or less), participants were again not solely using speed.

As the distance and duration were greater in the catch interval, we could infer which cue participants were using to complete the speed discrimination task.

*Speed:* If participants used only speed, not duration or distance, to make their judgement, they would pick the catch interval on 50% of occasions (as we used a forced-choice task and the speeds in each interval were identical). In this scenario, we would accept the null hypothesis, and performance would be consistent with participants using only speed information.

*Distance*: If participants used the distance cue to make their judgement, they would pick the catch interval significantly more than 50% of the time (because the distance travelled in the catch interval was further, and something that travels further may be thought of as travelling faster). In this scenario, we would reject the null hypothesis that the participant is using only speed information.

*Duration*: If participants used the duration cue to make their judgement, they would pick the catch interval significantly less than 50% of the time (because the standard interval had the shorter duration, and something that travels the same distance in a shorter duration may be thought of as travelling faster). Again, in this scenario, we would reject the null hypothesis that the participant is using only speed information.

### 2.5. Analysis

**Figure 3:**
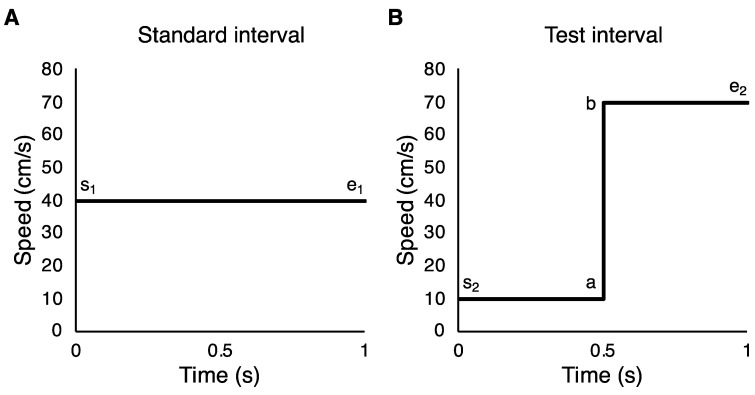
Speed-time graphs of (**A**) a standard interval and (**B**) a test interval in the *Speed change* condition. Participants could have used several different strategies to complete the speed change discrimination task—they could have compared points a and b, s_1_ and s_2_, or e_1_ or e_2_.

We conducted several different analyses, with the aim of understanding what cues, and hence what strategies, participants might be using to perform the task. We measured 75% thresholds for speed change discrimination and speed discrimination by fitting cumulative normal psychometric functions using MATLAB R2014b (The MathWorks Inc, Natick, MA, USA) and the Palamedes toolbox [24]. There were several different strategies that participants could have adopted to complete the *Speed change* condition (see Figure 3). Two specific strategies to compare are (i) using the speed change within intervals and (ii) using the speed difference between intervals at a specific point in time (e.g., the end of each interval). The speed change strategy (i) entails computing the speed change within each interval (e_1_–s_1_ versus e_2_–s_2_ or b–a) and comparing these quantities. The speed difference strategy (ii) requires comparing speeds between the intervals, for example the stimulus end speed for each interval (e_2_ versus e_1_), or the start speed (s_2_ versus s_1_).

In order to determine which of these strategies was better, we needed to compare the relative d’ difference between them. d’ is defined as the separation of means between signal and noise, divided by the standard deviation of the distributions. We can calculate this relationship by using the standard rules for propagation of uncertainty. We can illustrate this using simple quantities, and the relative relationships will hold for other values. For example, first let the b–a test interval be 1, and let the noise be uncorrelated Gaussian distributed with unit variance. The speed change strategy (i) requires calculating the double difference: (e_1_–s_1_) − (e_2_–s_2_). By the rules of propagation of uncertainty, each term adds 1 unit of variance to the final estimate, giving a variance of 4. The expected value of this double difference is equal to the b–a interval of 1, giving d’ = 1/√4 = 0.5.

The end speed or start speed difference strategy (ii) requires calculating the difference e_2_–e_1_ or s_2_–s_1_. Here, the separation between means is 0.5 because the standard speed is halfway between the extreme values. In both of these cases, two quantities are combined, each term adds 1 unit of variance for a total of 2. Thus, d’ = 0.5/√2 = 0.354. This demonstrates that the speed change strategy is a more optimal strategy by a factor of √2.

We therefore conducted our psychometric analysis for the *Speed change* condition in two different ways:

(I) We found the speed change within the test interval required to respond correctly on 75% of occasions as a function of world speed (cm/s).
(II) We measured 75% thresholds on the basis of the difference in speed between speeds at the end points of the standard and test *Speed change* intervals in cm/s.

For the *Distance* and *Duration* speed discrimination conditions, participants had to compare speeds between intervals to perform the task. This is akin to strategy (ii) for completing the *Speed change* condition above with the b-a difference separated across the two intervals instead of within 1 interval. Therefore, the d’ is calculated using an expected difference of 1, and results in a total variance of 2 as in strategy (ii), for a d’ of 1/√2 = 0.707. We therefore measured thresholds for these conditions on the basis of the difference in speed between the first and second interval, required to respond correctly on 75% of occasions. If participants were using strategy (ii) to complete the *Speed change* condition, we would expect thresholds calculated using analysis (I) to be a factor of 2 worse, and using analysis (II) to be similar to those in the *Duration* and *Distance* speed discrimination conditions.

**Figure 4:**
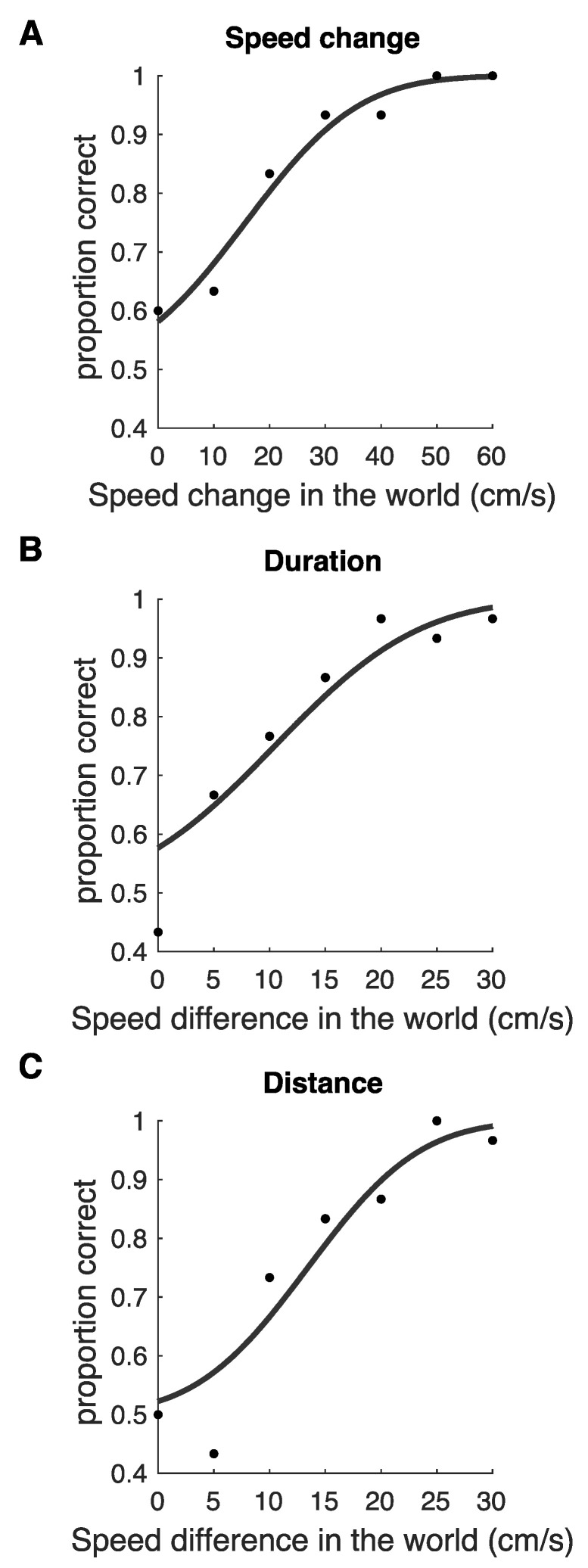
Example individual participant psychometric functions in the experimental conditions. (**A**) is a psychometric function from a participant in the *Speed change* discrimination condition, using analysis I. (**B**) is a psychometric function from the *Duration* speed discrimination condition, where duration information varied in the stimulus along with speed, but distance information was held constant. (**C**) is a psychometric function from the *Distance* speed discrimination condition, where distance information varied in the stimulus along with speed, but duration information was held constant. (**B**,**C**) are data from the same participant.

Example psychometric functions are shown for the *Speed change* condition (analysis I; Figure 4A), *Duration* condition (Figure 4B), and *Distance* condition (Figure 4C). We did not plot psychometric functions for the *Speed change* type II analysis in Figure 4. These psychometric functions use the same data as those for analysis I, but use the same *x*-axes as the *Distance* and *Duration* conditions.

JASP (Version 0.9.1; JASP Team, 2018) was used to conduct a pair of Bayesian and Frequentist two-sample *t*-tests. Threshold values between the *Speed change* discrimination condition and the *Duration* speed discrimination condition, and the *Speed change* discrimination condition and the *Distance* speed discrimination condition were compared. This was done to determine if there was a difference in threshold between the *Speed change* discrimination condition and the two speed discrimination conditions. A Frequentist and corresponding Bayesian paired *t*-test was then used to compare thresholds between the *Distance* and *Duration* speed discrimination conditions, to investigate if thresholds varied depending on whether duration or distance information were available, respectively. The significance level for all Frequentist *t*-tests was taken as 0.0167 (Bonferroni correction for 3 comparisons). For all Bayesian *t*-tests, the prior used was a Cauchy distribution centred on zero with a width of 0.707, which is the default prior for Bayesian *t*-tests in JASP Version 0.9.1. We also measured Pearson’s correlation coefficient between the catch trial results for each participant in each speed discrimination condition to observe whether individual participants consistently used the same cue to complete the catch trials in both the *Duration* and *Distance* conditions. When conducting Bayesian statistical analysis, we report either Bayes factors weighted towards the alternative hypothesis (BF_10_), or Bayes factors weighted towards the null hypothesis (BF_01_). For a review of the rationale of using Bayesian statistics, see [25].

## 3. Results

### 3.1. Comparing Speed Discrimination and Speed Change Discrimination

**Figure 5:**
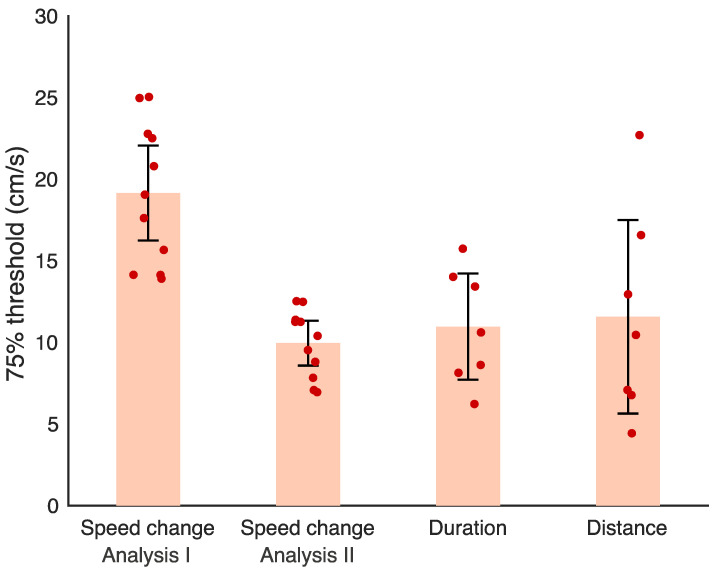
The 75% thresholds for the *Speed change* discrimination condition using both methods of analysis (*n* = 11), and the *Duration* and *Distance* speed discrimination conditions, both *n* = 7. Error bars are 95% confidence intervals. Individual data points for each condition are shown in red. *Speed change* analysis I was conducted in terms of the speed change within the test interval. *Speed change* analysis II was conducted in terms of the difference between the end speed of the test and standard intervals.

When the *Speed change* condition was analysed in terms of the speed change within the test interval (analysis method I), speed change discrimination was found to be more difficult than speed discrimination when duration or distance information was available (Figure 5). Speed change discrimination thresholds were significantly higher than those for speed discrimination with a fixed distance and variable duration (*Duration* condition; t(16) = 4.180, *p* < 0.001) and also were higher than for speed discrimination with a fixed duration and variable distance (*Distance* condition; t(16) = 3.009, *p* = 0.008). A Bayes factor (BF_10_) of 38.49 was found for the comparison between the *Speed change* condition and the *Duration* condition, which indicates very strong evidence that there was a significant difference between the *Speed change* and *Duration* conditions [26,27]. For the comparison between the *Speed change* and the *Distance* conditions, a Bayes factor (BF_10_) of 5.90 was found, indicating substantial evidence that there was a significant difference between the *Speed change* and *Distance* conditions [26,27]. There was no significant difference in thresholds between the *Duration* and *Distance* conditions (t(6) = -0.381, *p* = 0.716). A Bayes factor (BF_01_) of 2.66 was found for this *t*-test, indicating anecdotal evidence that there was no significant difference between the *Distance* and *Duration* conditions [26,27,28].

To investigate if participants were using a sub-optimal strategy to complete the speed change discrimination task, we additionally conducted psychometric analysis for the *Speed change* condition in terms of the difference in speed between the end point of the standard and test intervals within a *Speed change* trial (analysis method II). There was no significant difference between the *Speed change* condition and the *Duration* condition (t(16) = 0.778, *p* = 0.448), or the *Speed change* condition and the *Distance* condition (t(16) = 0.786, *p* = 0.443; see Figure 5). A Bayes factor (BF_01_) of 1.95 was found for the comparison between the *Speed change* and *Duration* conditions, while a Bayes factor (BF_01_) of 1.94 was found for the comparison between the *Speed change* and *Distance* conditions. Both of these Bayes factors indicated anecdotal evidence for the null hypothesis that there was no difference between the *Speed change* and speed discrimination conditions [26,27,28]. Outputs from the psychometric functions were identical for the *Distance* and *Duration* conditions using *Speed change* analysis methods I and II.

These results suggest that participants who completed the *Speed change* condition may have been using the speed at the end point, or the start point, of each interval within a trial to make their judgement, rather than identifying the change in speed within the test interval. As the standard interval in the *Speed change* condition contained a stimulus that always travelled at a constant 40 cm/s, while the test interval contained a stimulus that travelled slower and then faster than this speed, comparing the start or end points of the two intervals within a trial represents a sub-optimal strategy for completing the task. The difference in speed within the test interval was always greater than the difference between the end points between the standard and test intervals, and yet the difference in speeds between the start and end point of the test interval appears to have not been used.

### 3.2. Cue Usage in Catch Trials

**Figure 6:**
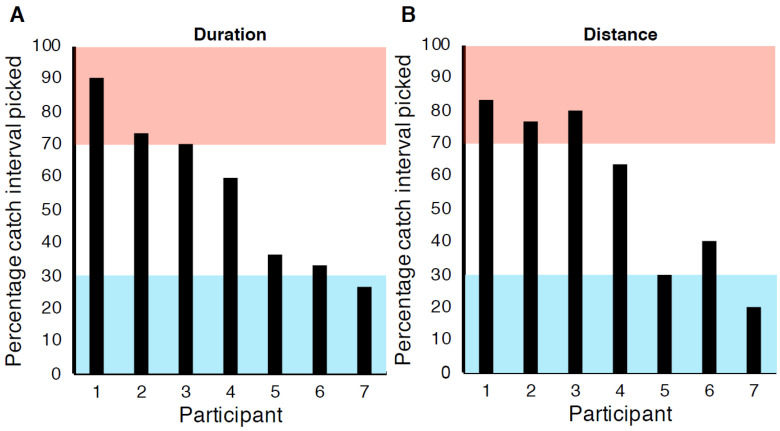
The percentage of occasions the catch interval was picked over the standard interval in the catch trials for (**A**) the *Duration* speed discrimination condition and (**B**) the *Distance* speed discrimination condition. Participants whose data lie in the pink region used distance cues. Within the blue region, participants used duration information. Data in the white region suggest participants used speed.

Our catch trial analysis showed that participants used a variety of strategies to complete the task (Figure 6). We categorised the catch trial results into three groups on the basis of binomial proportion confidence intervals:

(I) 0–30% catch interval picked—We reject the null hypothesis that participants used only speed. Participant was using duration as a cue.
(II) 70–100% catch interval picked—We reject the null hypothesis that participants used only speed. Participant was using distance as a cue.
(III) 31–69% catch interval picked—We do not reject the null hypothesis. Participant was primarily using speed to make their judgements in the catch trials.

From the pattern of data in Figure 6A, it appeared that in the *Duration* speed discrimination condition, one participant used the shorter duration information, three participants used speed information, and three participants used the longer distance information to complete the task. In the *Distance* speed discrimination condition, two participants used the shorter duration, two participants used speed information, and three participants used the longer distance information to complete the task (see Figure 6B). Our data suggest that different participants used different cues to complete speed discrimination tasks.

### 3.3. Individual Participant Data

Given that some individual participants appeared to use cues other than speed in the catch trials, we might expect that individual participants would perform better in the main experiment condition where they could use the cue they favoured in the catch trials. For example, we might predict that participant 1 in Figure 6, who appeared to use distance information to make their catch trial judgements, would have improved performance in the *Distance* condition of the main experiment, as they could use their favoured cue from the catch trials. In the *Duration* condition of the main experiment, we might predict worse performance from participant 1, as there was no distance information available for them to use.

**Figure 7:**
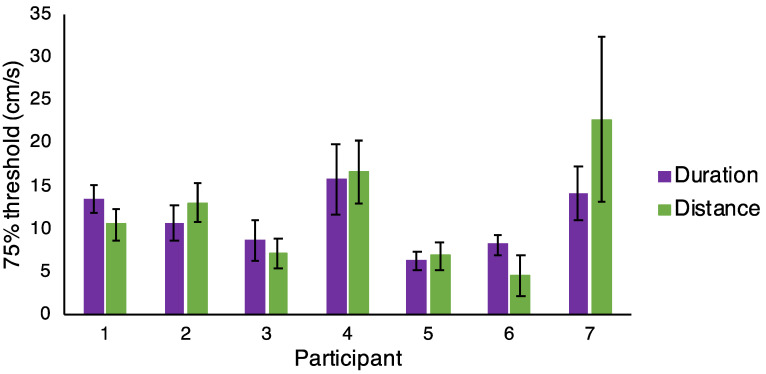
Individual 75% thresholds for each participant in the two speed discrimination conditions (*Duration* and *Distance*, *n* = 7)**.** Error bars are standard error from the psychometric fit.

There were marked individual differences in performance, as has been found previously in experiments involving speed discrimination that have used naïve observers [29]. However, individual participants did not show a pattern of thresholds in the main experiment that would be predicted from their behaviour during the catch trials. Figure 7 illustrates that there was, for all participants, very little difference in threshold between the two speed discrimination conditions in the main experiment.

### 3.4. Did Participants Use the Same Cues in the Catch and Main Experiment Trials?

Notice that individual participants were consistent in their choice of catch trial cue usage between the two catch conditions (Figure 6). A significant strong correlation of percentage catch interval picked per participant was found between the two catch conditions (*r* = 0.963, *n* = 7, *p* < 0.001, BF_10_ = 51.02). This suggests participants may have been using cues to perform the task in the catch trials that would not be helpful if used in the main experimental trials. For example, three participants appeared to use distance information in the catch trials included as part of the *Duration* speed discrimination condition (see Figure 6A). In this *Duration* condition, speed and duration cues were available but the distance travelled was fixed. This means those participants used distance information in the catch trials despite it being impossible to successfully use distance to make judgements in the main experiment trials of this condition. As the catch trials were interleaved with the main experimental trials—This suggests that participants may have been changing between using different cues from one trial to the next when completing the speed discrimination task. Our design was not aimed at testing whether participants used a mixture of cues, so we were unable to investigate this further.

## 4. Discussion

The aims of this work were to first determine if speed change discrimination for looming stimuli is a more difficult task than speed discrimination, and second, to investigate if participants use distance or duration information, instead of speed information, when it is available in speed discrimination tasks. If so, this might explain the apparent difficulty of speed change discrimination judgements. To do this, we measured discrimination thresholds for the two different tasks and included manipulations of the speed discrimination stimuli in the form of “catch trials”, which were designed to reveal the use of distance, duration, or speed information.

When analysing our *Speed change* data in terms of the speed change within a test interval, we found speed change discrimination to be significantly more difficult than speed discrimination for looming stimuli. This result is in agreement with the previous literature that has studied speed change discrimination for 2D motion and other varieties of 3D motion [11,16,20]. However, our results were consistent with participants comparing the start or end speeds of the standard and test intervals in the *Speed change* condition, rather than judging the speed change. This was surprising because, as described in the analysis section of the Methods section, judging speed change at the change-point would be expected to deliver a d’ improvement of √(2) over other strategies. This may suggest that participants found judging the speed change within the test interval so difficult that they did not use it. If participants did not use the speed change to make their judgements, instead comparing start or end speeds between intervals, the lack of distance and duration cues in the speed change discrimination task likely does not explain why these tasks have appeared more difficult in previous studies (e.g., [11,16,20]).

In experiments from other labs using a different type of speed change discrimination task, where a participant determined whether a stimulus has increased or decreased in speed, the distinction between speed change and speed discrimination thresholds was again less clear. Thresholds have been reported to be roughly three times higher when two speeds are presented consecutively than when there was a period of 1 s between the two speeds, but only when the duration of motion was shorter than that which we used in our experiment [30]. In another study, thresholds as low as 12% were found for this type of experiment [31]. However, unlike in the two-interval forced choice speed change discrimination task used in our study, Hick’s [31] experimental setup left it clear when a speed change had occurred, which could have made their task easier and thus would explain their low thresholds.

Our manipulations of the catch trials in the speed discrimination task suggested that participants were not exclusively using speed to complete the task. We found some participants who appeared to use speed, some duration, and some distance in the catch trials, and the cue use correlated between the catch trials in the *Duration* and *Distance* conditions. This means in some cases participants would have used catch trial strategies that they could not have used successfully for other trials in the main experiment. For example, in Figure 6, participant 1 appeared to often be using the distance cue to make their judgements in the catch trials in both the *Duration* condition (Figure 6A) and the *Distance* condition (Figure 6B). However, participant 1 would not have been able to successfully use distance to perform the speed discrimination task in the main experiment trials of the *Duration* condition, as the distance cue was not informative. As participant 1 was able to perform this task well (see Figure 7), we can infer that participants may have been able to use multiple cues to complete the speed discrimination task, and, as the catch trials were randomly interleaved amongst the other experimental trials, that participants could have been switching between using different cues from trial to trial. It is also possible that participants may have used a combination of differently weighted cues to make their judgements in each trial, or that participants may have used different cues more on different days, but our design was not aimed at testing these hypotheses. As such, we cannot address this possibility here.

The finding that participants may have used cues other than speed to complete the speed discrimination task is supported by other studies that have shown that participants may not use speed information in isolation to discriminate speed [32,33]. Mandriota et al. [32] found that adding distance and duration cues improved speed discrimination performance, whilst Smith and Edgar [33] reported that discrimination of speed and temporal frequency were inseparable when using drifting grating stimuli, suggesting speed may not be used in isolation.

On the other hand, our catch trial findings conflict with research that suggests that participants use only speed in speed discrimination tasks with both 2D and 3D motion [2,12,14,18,34,35]. However, of these studies, only Lappin et al. [34] included more than three human participants in their study, although Pasternak [35] also had nine cats as subjects. In our study, all participants were fully naïve and inexperienced observers. Three of these previous studies had at least one author as a participant who would have been aware of the aims of the experiment [2,18,35]. It is possible that if a larger number of naïve participants had been used in these experiments, a greater range of cue usage in speed discrimination tasks may have been demonstrated.

In summary, we found that participants did not always use speed information in speed discrimination tasks, instead using distance or duration cues, and that participants appeared to not use the speed change within in a speed change discrimination task. Participants instead appeared to use a sub-optimal strategy and compared start or end points of the two intervals to make their speed change judgements. This suggests that making a judgement about the speed change may have been so difficult that observers did not use that information. Why the change in speed was not used, when the speed change *within* a test interval was greater than the difference in start or end speeds *between* intervals, is an interesting avenue for future research.
